# Functional characterization of the domains of the bovine binder of SPerm 5 (BSP5) protein

**DOI:** 10.1186/s12958-015-0058-4

**Published:** 2015-06-19

**Authors:** Prashanth Sirigeri Jois, Geneviève Plante, Isabelle Thérien, Puttaswamy Manjunath

**Affiliations:** Maisonneuve-Rosemont Hospital Research Centre, Montreal, QC Canada; Department of Biochemistry and Molecular Medicine, Faculty of Medicine, University of Montreal, Quebec, Canada; Department of Medicine, Faculty of Medicine, University of Montreal, Quebec, Canada; Maisonneuve-Rosemont Hospital Research Center, 5415 l’Assomption blvd, Montreal, QC H1T 2 M4 Canada

**Keywords:** BSP5, Fibronectin type II domain, Escherichia coli expression system, Recombinant domains, Bovine sperm, Capacitation

## Abstract

**Background:**

Bovine BSP5 is a multifunctional protein primarily involved in sperm capacitation. BSP5 consists of long N-terminal part followed by two similar and highly conserved fibronectin type II domains designated A and B.

**Methods:**

In order to assess the role of these domains in the sperm binding and capacitation processes, we created recombinant individual domains (N, A, B), series of overlapping domains (NA and AB) and full-length BSP5 in an Escherichia coli expression system. The recombinant constructs were also tested for their ability to interact with ligands such as gelatine, heparin, chondroitin sulphate B and phosphatidylcholine liposomes by affinity chromatography and co-sedimentation studies.

**Results:**

With the exception of the N domain, all recombinant constructs retained gelatine, phosphatidylcholine, heparin and chondroitin sulphate B binding activities. Domain-wise studies showed clearly that AB domain is capable of performing its biological functions as well as the full-length protein, as it was able to potentiate heparin-mediated sperm capacitation.

**Conclusions:**

These results indicate that the C-terminal domain composed of two Fn2 domains is sufficient and crucial to maintain the biological functions of BSP proteins. The N-terminal part of the protein did not bind to any of known BSP5-ligands including epididymal sperm and did not seem to be required for either sperm binding or sperm capacitation. This study also confirmed that glycosylation is not required for BSP-mediated sperm capacitation or any of the binding characteristics displayed by BSP5.

## Background

Seminal plasma is a complex mixture of secretions from testes, epididymides and accessory sex glands. Several components of mammalian seminal plasma are involved in the membrane remodeling processes occurring during sperm maturation [1]. Among these, proteins of Binder of SPerm (BSP) superfamily, previously called Bovine Seminal Plasma proteins, are the most studied in terms of biochemical properties, structure and functions [2].

The BSP proteins all have a common structure composed of a variable N-terminal part followed by two tandem fibronectin type-II (Fn2) domains characterized by the presence of several highly-conserved aromatic residues and four invariant cysteine residues forming intra-domain disulfide bonds with the connectivities 1–3 and 2–4. Initially discovered in the bovine seminal plasma, BSP protein homologs have since then been purified and characterized from seminal plasma of boar, goat, ram, bison, buffalo and stallion [3–9]. BSP homologous DNA sequences have also been identified in the genomes of human, mouse, rat, dog and chimpanzee; and BSP-like epididymal proteins have been identified in rabbit, human and mouse [10–14].

In bovine species, three proteins BSP1, BSP3 and BSP5 (previously called PDC-109 or BSP-A1/A2, BSP-A3 and BSP-30 kDa respectively) have been purified and biochemically characterized [15, 16]. The three proteins are glycosylated to various extents, BSP5 being the most glycosylated and BSP3 having no glycosylation at all. They are known to bind to sperm upon ejaculation via an interaction with choline phospholipids. Interestingly, as opposed to BSP1 and BSP3, which binds specifically to choline phospholipids, BSP5 has been shown to display a broader binding specificity to lipids [17]. Some studies performed with BSP1 demonstrated that its binding to phosphatidylcholine (PC) liposomes and to bovine epididymal sperm membrane is a very rapid, biphasic process [18]. In the first step, the BSP protein recognizes and interacts with the choline head group of the phospholipid. This causes a conformational change in the protein, which enables the penetration of short segments of the protein in the hydrophobic interior of the membrane up to the fourteenth atom of carbon of the phospholipid [19–21]. When BSP proteins are in low concentrations, this interaction ultimately results in the rigidification of the lipid phase [18, 22].

Through this interaction with phospholipids, BSP proteins also play dual roles in bovine sperm capacitation, a complex series of molecular modifications that mammalian sperm must undergo before fertilizing an oocyte. Via membrane stabilization, BSP proteins act as decapacitating factors. They protect sperm during their transit through the female genital tract following ejaculation and prevent premature sperm capacitation. Bovine BSP proteins also promote sperm capacitation by inducing cholesterol and phospholipid efflux from sperm [23, 24]. They can induce this efflux when they are found in high concentrations or through interactions with high-density lipoproteins and glycosaminoglycans (GAGs) such as heparan sulphate found in follicular fluid [25, 26].

BSP proteins in bovine species bind to diverse ligands such as gelatine [15], heparin [27], other GAGs [28] and apolipoprotein A-I [29]. They also bind to components of semen extenders namely, egg yolk-low density lipoproteins and major milk proteins (caseins, α-lactalbumin and β-lactoglobulin) used for semen preservation. This interaction is important for sperm protection [30] [31]. The mechanisms to regulate and coordinate the interaction of BSP proteins with diverse set of ligands in order to accomplish function(s) are not clear. In addition to their role in sperm capacitation, other functions have been proposed for bovine BSPs such as the mediation of sperm binding to the oviductal epithelium, the formation of the sperm reservoir and the ability to prolong sperm survival and motility in the oviduct [32, 33]. Recent studies also reported that bovine BSP1 can function as a molecular chaperone *in vitro* [34].

So far, most of the knowledge on BSP proteins has been obtained from the study of bovine BSP1, as this protein is found in the highest concentration in bovine seminal plasma [35]. However, BSP5 is known to have the longest N-terminal domain, to be the most glycosylated and to be the most active member of the bovine BSP proteins [26, 25]. Very few studies have been done to understand if the individual domains in multi-domain proteins such as BSP5 have ligand binding specificity and exercise biological function(s). The importance of the glycosylation in BSP function(s) and the minimal functional unit required to mediate the sperm capacitation are also not known.

In the current study we successfully cloned, expressed and purified domains (N, A and B), linear combination of domains (NA and AB) and the full length BSP5 employing *Escherichia coli* as host. We then analyzed the ligand binding properties and the biological activities of individual and overlapping recombinant domains of bovine BSP5.

## Methods

Plasmids pET32a(+) and pET30(+), *Escherichia coli* strains BL21(DE3)pLysS and Origami B(DE3) and His-bind resin were from Novagen (EMD Biosciences, La Jolla, CA, USA). *Pfu* DNA polymerase was from Fermentas (Burlington, ON, Canada) and *Taq* DNA polymerase was from GE Healthcare (Baie d’Urfe, QC, Canada). Restriction enzymes *BamHI* and *XhoI* were from New England Biolabs (Beverly, MA, USA). QIAprep Spin Miniprep kit and Qiaex II gel extraction kit were from Qiagen (Mississauga, ON, Canada). T4 DNA ligase was from Invitrogen (Carlsbad, CA, USA). For Western blotting, the His-probe monoclonal antibody (H-3) was from Santa Cruz Biotechnology (Santa Cruz, CA, USA), whereas goat anti-mouse IgG and goat anti-rabbit IgG were from Bio-Rad (Mississauga, ON, Canada). Chemiluminescence reagent was from Perkin-Elmer (Boston, MA, USA). Complete Mini, EDTA-free protease inhibitor tablets were from Roche (Manheim, Germany). B-PER bacterial extraction reagent was from Pierce (Rockford, IL, USA). Bovine serum albumin (BSA; fraction V fatty acid-free), heparin (purified from porcine intestinal mucosa), taurine, L-epinephrine, erythrosin B, flavianic acid (naphthol), lyso-phosphatidylcholine (lyso-PC; purified from egg yolk) were from Sigma-Aldrich Canada (Oakville, ON, Canada). Penicillin G and streptomycin sulphate were from Gibco (Burlington, ON, Canada), eosin B from Fisher Scientific (Ottawa, ON, Canada) and nigrosin from Kodak (Rochester, NY, USA). All other chemicals used were of analytical grade and obtained from commercial suppliers.

### Construction of BSP5 expression vectors

To generate expression vectors for the production of full-length bovine BSP5 (FL; residues 1–158), individual domains (N, A and B) and linear combination of domains (NA and AB), corresponding regions of the *BSP5* gene were amplified by polymerase chain reaction using bovine seminal vesicle cDNA as template. The amplified DNA of the N domain was inserted into a pET30a vector and expressed in *E. coli* BL21(DE3) cells as described previously [36]. All the other domains were cloned in pET32a vectors and were expressed in *E. coli* Origami B(DE3) cells. As control, the empty pET32a vector was transformed in *E. coli* BL21(DE3) cells to express the thioredoxin-His-S tag (Trx-His-S).

### Expression and purification

Bacteria were grown at 37 °C in Luria–Bertani medium containing either ampicillin (100 μg/ml) or kanamycin (50 μg/ml) until OD_600nm_ reached ~ 0.8. Induction was then carried out with 100 μM isopropyl-β-d-galactopyranoside (IPTG) at 15 °C for 16 h. For the FL protein, the individual domains (A and B), the linear combination of domains (NA and AB) and the Trx-His-S control, harvested cells were lysed by sonication in 50 mM Tris–HCl (pH 7.9), 150 mM NaCl, 40 mM imidazole buffer, containing 10 % B-PER (v/v), and protease inhibitor cocktail. They were then purified by Ni-NTA affinity chromatography according to the manufacturer’s instructions. Proteins were eluted from the column using buffer containing 250 mM imidazole.

As the N-terminal part was sensitive to protease, boiling lysis instead of sonication was used to purify the protein as previously described [36]. Briefly, the cell suspension was incubated in boiling water for 20 min, cooled on ice for 5 min, and centrifuged at 4 °C, at 15 000 rpm for 1 h. The supernatant was purified by Ni-NTA affinity chromatography similar to the other constructs. The purity of each construct was examined by SDS-PAGE. Five micrograms of each sample were separated on a 15 % polyacrylamide gel followed by Coomassie Brilliant Blue staining. Fractions containing purified protein were pooled, dialysed against 50 mM ammonium bicarbonate and lyophilized.

The native BSP5 protein used as control in the experiments was purified from bovine seminal plasma by gelatine-agarose affinity chromatography as previously described [15]. The protein estimations were done by a modified Lowry method [37]. Lyophilized protein samples were weighed in the microbalance (Cahn C-31) and appropriate quantities were used for the subsequent studies.

### Affinity chromatography

All the binding studies by affinity chromatography were performed at 4 °C at a flow rate of 20 ml/h. For the binding to gelatine, five hundred micrograms of the lyophilized recombinant constructs and Trx-His-S were solubilised in 5 ml of TB (Tris-Buffer; 50 mM Tris–HCl, pH 7.4) and centrifuged 10 000 × *g* for 10 min to remove any insoluble material. The supernatant were then applied on a gelatine-agarose column (bed volume of 2 ml) previously equilibrated with TB. The column was washed with 10 bed volumes of TB and adsorbed proteins were eluted with TB containing 8 M urea.

For the binding to glycosaminoglycans, 1 mg of recombinant BSP5 protein constructs in 1 ml of the TB containing 40 mM NaCl was applied to a heparin-sepharose or a chondroitin sulphate B-agarose column (bed volume, 1 ml) equilibrated in TB containing 40 mM NaCl. The columns were washed with 10 bed volumes of the same buffer and the adsorbed material was eluted with TB containing 1 M NaCl and with the TB containing 8 M urea. For all the chromatography assays, fractions of 1 ml were collected and protein elution was monitored by optical density measured at 280 nm. Proteins from each peak were pooled, concentrated by ultrafiltration, precipitated with 15 % trichloroacetic acid (TCA) and analyzed by 15 % SDS-PAGE followed by staining with Coomassie Brilliant Blue.

### Lipid vesicle co-sedimentation assay

Liposomes of phosphatidylcholine (PC) were prepared as described in [17]. Briefly, 8 mg of PC (Doosan Sedary Research Laboratories; Englewood Cliffs, NJ, USA) in chloroform was evaporated under N_2_ until a thin film was formed in the bottom of a glass tube. PC was then resuspended in 2 ml buffer (10 mM Tris–HCl, 100 mM KCl, pH 7.5) and the tube was sonicated in a Branson Ultrasonic water bath (Model 3510) for ~1 min at RT until lipids formed an opalescent suspension. Large multi-lamellar liposomes were sedimented by ultracentrifugation at 100 000 × *g* at 25 °C for 30 min. The pellet containing liposomes was resuspended in buffer B (10 mM Tris–HCl, 100 mM KCl, 2.5 mM MgCl_2_, pH 7.5), to obtain a final lipid concentration of 4 mg/ml. To test the binding to PC liposomes, 20 μg of recombinant BSP5 constructs, 10 μg of Trx-His-S and 10 μg of BSP5 were incubated with liposomes (equivalent to 150 μg PC), in a total volume of 300 μl of buffer B. Incubations were carried out for 30 min at RT, after which liposomes were centrifuged once again at 100 000 × *g* at 25 °C for 45 min. Equivalent fractions of supernatant (proteins precipitated with 15 % TCA) and pellet were analyzed by 15 % SDS-PAGE followed by Coomassie Brilliant Blue staining and by Western blot.

### Sperm binding assay

Bovine epididymides were obtained from a local slaughterhouse near Montreal. Cauda sperm collected from the epididymides were washed twice (300 × *g*, 10 min) with 10 volumes of a modified Tyrode’s medium designated mTALP (100 mM NaCl, 2.7 mM KC1, 1.8 mM CaCl_2_, 0.5 mM MgCl_2_, 0.3 mM NaH_2_PO_4_, 35.7 mM NaHCO_3_, 0.1 mM sodium pyruvate, 17.4 mM sodium lactate, 0.5 mM taurine, 0.05 mM L-epinephrine, 63 μg/ml penicillin G (1585 U/mg), 0.1 mM streptomycin sulfate, and 20 μg/ml phenol red dye) [26]. Washed sperm (10 × 10^6^ cells) were then incubated in 1 ml mTALP for 90 min at 39 °C with 20 μg/ml of respective recombinant BSP5 construct, 20 μg/ml Trx-His-S or 20 μg/ml native BSP5. Following the incubation, sperm were pelleted at 5000 × *g* for 10 min. The supernatant was removed and the pellet was washed three times with 1 ml of PBS. The supernatant was precipitated with TCA (15 %, final concentration), resuspended in Laemmli sample buffer and boiled 10 min. The pellet was resuspended in sample buffer, boiled 10 min and sonicated 1 h in a water bath. Supernatants and pellets were finally analyzed by western blot.

### Sperm capacitation and acrosome reaction

Epididymal sperm were prepared as described above. Washed sperm (final concentration of 5 × 10^7^ cells/ml) were preincubated (5 % CO_2_, 39 °C) for 20 min in the presence or absence of different concentrations of each domain constructs. Sixty μg/ml Trx-His-S and 30 μg/ml native BSP5 were used as negative and positive control respectively. The cells were then washed twice (300 × *g*, 10 min) and resuspended (final concentration of 5 × 10^7^ cells/ml) with 12 μg/ml of heparin. After the 5 h incubation, two aliquots of each sample were taken. The first aliquots were re-incubated for 15 min in the absence (spontaneous acrosome reaction) or in the presence of 100 μg/ml of lyso-PC. This concentration of lyso-PC was previously shown to induce the acrosome reaction in capacitated sperm while having no effect on noncapacitated sperm [38]. The other sperm aliquots were used for immunofluorescence study.

### Staining procedure

To assess acrosome reacted status of the bovine sperm, the naphthol yellow S-erythrosin B staining by Lenz et *al.* was used [39]. Briefly, the slides were placed in 0.1 % naphthol yellow S in 1 % aqueous acetic acid for 30 min and rinsed in 1 % aqueous acetic acid for 10 s. The slides were then drained, placed in a solution of 0.2 % aqueous erythrosin B and 0.2 % naphthol yellow S (pH 4.8) for 13 min, rinsed in distilled water, and air dried.

### Sperm viability

Sperm viability was estimated by the staining protocol of Dott and Foster [40]. Briefly, an aliquot of each sperm suspension (10 μl) was mixed with 5 μl of eosin B 5 %, and 5 μl of a saturated solution of nigrosin was added. The stained sperm were spread on glass slides, dried. Viable (white) and non-viable (red) sperm were counted under light microscopy in duplicate.

### Immunofluorescence microscopy

Following capacitation in the presence of heparin, sperm incubated with 60 μg/ml of respective recombinant BSP5 construct, 60 μg/ml Trx-His-S or 30 μg/ml native BSP5 were used for immunofluorescence. Aliquot of the sperm suspension (10 μl) were mixed with 40 μl of PBS and 50 μl of 4 % paraformaldehyde to fix the sperm for 30 min at RT after which 450 μl of PBS were added. The sperm were pelleted at 2000 × *g* for 10 min, washed with 500 μl of PBS and resuspended in a final volume of 20 μl of PBS. Two aliquots of 5 μl of the sperm suspension was applied to Poly-L-lysine slides and allowed to dry on a slide warmer. Sperm were permeabilized for 5 min with PBS containing 0.1 % Triton-X-100 and 0.2 % paraformaldehyde, and then washed 3 times with PBS. Nonspecific binding sites were blocked by incubating slides for 1 h at RT in PBS containing 1 % of BSA. Slides were allowed to dry and then incubated 1 h at RT with anti-BSP5 polyclonal antibodies or with His-probe mouse antibodies (1:100) [16].

Slides were washed 3 times with PBS for 5 min. Slides were allowed to dry and incubated at RT in a dark chamber for 1 h with the secondary FITC-labelled goat anti-rabbit IgG or secondary FITC-labelled goat anti-mouse IgG (1:200) in PBS-0.1 % BSA. Slides were washed 3 times for 5 min in PBS and allowed to dry. Finally, a drop of 1.5 % DABCO (10 ml of 1,4-diazabicyclo[2.2.2]octane 15 % and 90 ml of glycerol) was deposited on the slides. As controls for the specificity of the immunofluorescence, sperm without any added proteins were fixed and incubated with pre-immune sera and FITC-labelled secondary antibodies or with FITC-labelled secondary antibodies alone. Fluorescence was visualized with a Leica DMRE microscope, and the data were acquired using a RETIGA EX digital camera (QIMAGING) coupled with OpenLab 3.1.1 software (OpenLab).

### Statistical analysis

The data were analyzed for significant differences by one-way covariance analysis followed by LSD multiple comparison tests or by a Student *t*-test on paired observations using GraphPad Prism 5 software (GraphPad, San Diego, CA, USA).

## Results

### Expression and purification of recombinant constructs

**Fig. 1:**
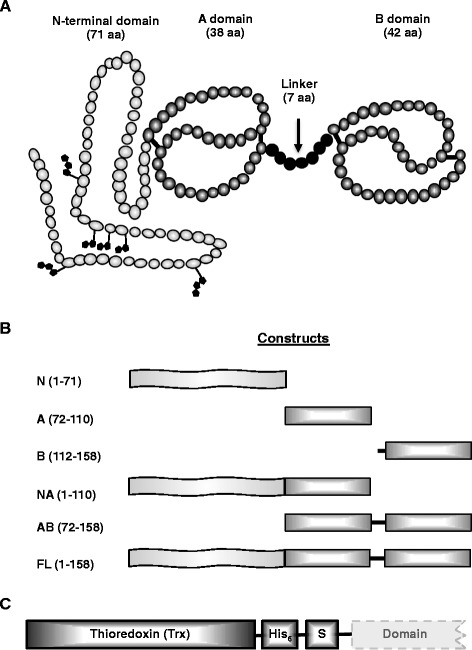
Schematic representation of bovine BSP5 and the recombinant constructs. **a** Native full-length bovine BSP5 is a 158 amino acid-protein composed of an N-terminal part and two Fn2 domains (**a** and **b**) joined by a short linker. BSP5 is glycosylated on six residues of its N-terminal domain. **b** Recombinant constructs of the BSP5 proteins used in this study. **c** The tag encoded by the pET32a(+) expression vector consists of a thioredoxin, followed by a (His)_6_-tag and a S-tag

BSP5 is a 158 amino acid-protein composed of a highly glycosylated N-terminal part and two Fn2 domains separated by a 7 residues-linker (Fig. 1a). In the current study, six different recombinant constructs were cloned and expressed in order to analyze the ligand binding properties and the biological activities of individual and overlapping recombinant domains of bovine BSP5 (Fig. 1b). Cloning, expression and purification of the N-terminal domain has already been described previously [36]. Since this domain does not contain any disulphide bonds in its structure, it was cloned in a pET30a vector and is fused only to a (His)_6_-tag. To help with the formation of the Fn2 domains and with protein refolding, the other five constructs were expressed in pET32a expression vectors and were therefore fused with a larger tag composed of a thioredoxin tag, a (His)_6_-tag and a S-tag (Fig. 1c).

**Fig. 2:**
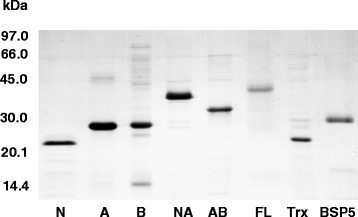
SDS-PAGE pattern of purified BSP5 recombinant constructs. pET32a(+) constructs of full length (FL), A domain (A); B domain (B); NA domain (NA) and (AB) domain were expressed in E. coli origami(DE3) cells and purified by Ni^2+^-NTA affinity chromatography. The pET30a(+) N-terminal (N) construct was expressed in E. coli BL21(DE3) cells. Five micrograms of purified proteins were analyzed by 15 % SDS-PAGE followed by Coomassie Blue staining

Trx-His-S tagged domains were expressed in *E. coli* BL21(DE3) cells and purified by Ni^2+^-NTA affinity chromatography. All purified domains were then analyzed by SDS-PAGE on 15 % polyacrylamide gels stained with Coomassie Brilliant Blue. Constructs were more than 90 % pure (Fig. 2). The two controls, Trx-His-S and native BSP5, were shown to have similar levels of purity. Recombinant constructs N, A, B, NA, AB and FL were found to have apparent molecular weights of approximately 24, 29, 29, 32, 39 and 42 kDa respectively as previously reported. Identity of the constructs was also confirmed by Western blot using anti-BSP5 polyclonal antibodies or His-probe monoclonal antibody (not shown).

### Binding properties

Interaction of the recombinant constructs with gelatine, heparin and chondroitin sulfate B (CSB) was tested by affinity chromatography (Table 1). When less than five percent of the proteins were found in the adsorbed fraction, binding was considered as non-specific as was the case for the N domain as well as the negative control Trx-His-S. All the other constructs were found to interact with the ligands with varying affinities. As was previously observed for the native BSP5, the FL construct displayed a strong affinity for both gelatine and GAGs. Similar results were obtained with domains B and AB. In the case of domains A and NA, they showed strong affinity for heparin and CSB, but a slightly higher amount of proteins were found in the unabsorbed fraction.

**Table 1:** Summary of binding studies of recombinant BSP5 constructs by affinity chromatography

Constructs	Gelatine	Heparin	Chondroitin Sulfate B
N	-	-	-
A	++	++++	+++
B	+++	+++	+++
NA	++	++++	+++
AB	+++	++++	+++
FL	+++	+++	++++
Trx-His-S	-	-	-

(−) <5 % binding, (+) 5-25 %, (++) 26-50 %, (+++) 51-75 %, (++++) 76-100 %

Co-sedimentation was used to test the interaction of recombinant constructs with both PC liposomes and bovine epididymal sperm (Table 2). Binding was scored following SDS-PAGE and western blot when a band corresponding to the recombinant domain was visible in the pellet fraction containing the liposomes or epididymal sperm. Based on these criteria, similar results were obtained for binding to PC liposomes and to epididymal bovine sperm. All domains with the exception of N-domain were found to interact with PC liposomes and epididymal sperm. Trx-His-S and N-domain were only found in the supernatant fraction suggesting a lack of interaction. As control, recombinant domains were also incubated in the absence of liposomes and centrifuged. Under those conditions, all domains were found in the supernatant fraction only (data not shown).

**Table 2:** Summary of binding studies of recombinant BSP5 constructs to PC-liposomes and epididymal sperm by co-sedimentation

Constructs	PC-liposomes	Epididymal sperm
N	-	-
A	+	+
B	+	+
NA	+	+
AB	+	+
FL	+	+
Trx-His-S	-	-

(−) No binding, (+) Binding observed

### Heparin-induced capacitation assay

**Fig. 3:**
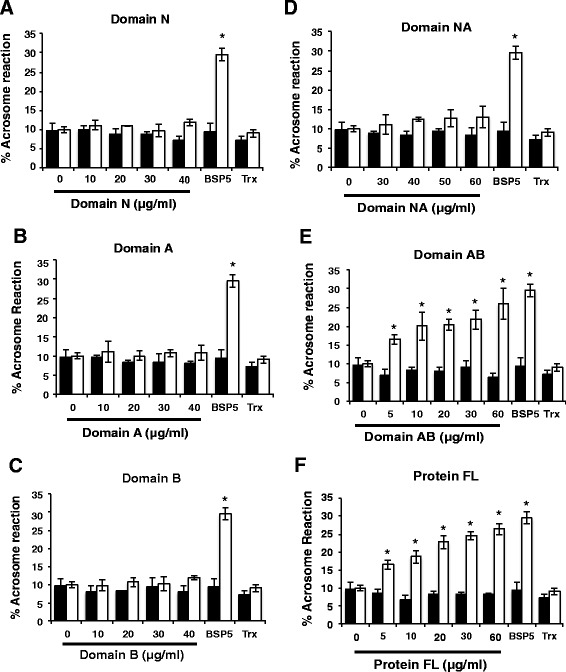
Effect of recombinant BSP5 constructs on lyso-PC-induced acrosome reaction of epididymal sperm incubated with heparin. Sperm collected from cauda epididymis were incubated for 20 min alone or in media containing increasing concentrations of the different BSP5 constructs, 60 μg/ml Trx-His-S or 30 μg/ml native BSP5. Sperm were then washed, incubated with 12 μg/ml of heparin for 5 h and incubated an additional 15 min in presence (white bars) or absence (black bars) of 100 μg/ml lyso-PC. Acrosome reaction was assessed by naphtol yellow S-erythrosin B staining. Data are presented as the mean ± SD of three independent experiments. Differences compared to control (no proteins added) were analyzed by one-way covariance analysis followed by LSD multiple comparison tests (* *p* < 0.05). Panels (**a**-**f**) correspond respectively to Domain N, Domain A, Domain B, Domain NA, Domain AB and Protein FL (Full length)

To examine whether the recombinant constructs of BSP5 were able to mediate heparin sperm capacitation, capacitation assays were carried out with different concentrations of the constructs, native BSP5 serving as positive control and Trx-His-S as negative control. Capacitation was assessed by the ability of sperm to undergo the AR induced by lyso-PC. Recombinant domains N, A, B and NA did not cause any significant increase in the percentage of acrosome reaction observed following incubation of sperm in the presence of lyso-PC (Fig. 3a-d; white bars). Increasing concentrations of the recombinant AB and FL constructs however caused a significant dose-dependent increase in the percentage of sperm acrosome reaction induced by lyso-PC, with a protein concentration as low as 5 μg/ml. Results obtained with AB and FL constructs were comparable to those to the native BSP5 positive control (Fig. 3e-f; white bars). Recombinant domains did not affect spontaneous acrosome reaction (without induction by lyso-PC), as after 5 h incubation with different domains, levels of acrosome reaction were equivalent to that of sperm incubated with media alone (Fig. 3; black bars).

### Sperm viability

**Fig. 4:**
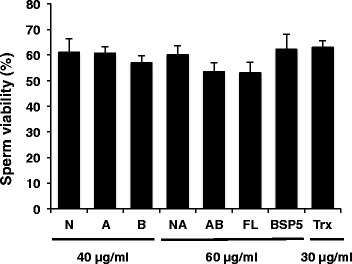
Effect of recombinant BSP5 constructs on the viability of epididymal sperm incubated with heparin. Sperm collected from epididymis were incubated for 20 min alone or in media containing 40 μg/ml of constructs N, A and B, 60 μg/ml of constructs NA, AB and FL, 60 μg/ml Trx-His-S or 30 μg/ml native BSP5. Sperm were then washed, incubated with 12 μg/ml of heparin for 5 h. Viability was assessed by staining with eosin/nigrosin solutions. Data are presented as the mean ± SD of three independent experiments

During the capacitation studies, the effect of recombinant proteins on sperm viability was also evaluated. None of the recombinant constructs including Trx-His-S affected the viability of the sperm (Fig. 4). At the beginning of the incubation with heparin approximately 75 % of sperm were viable. A slight decrease was observed toward the end of the incubation, but this effect was observed for all conditions tested.

### Immunolocalization of recombinant BSP5 constructs on bovine epididymal sperm

Binding of constructs to epididymal sperm was also confirmed by immunolocalization studies as signals were observed on epididymal sperm for all recombinant constructs except the N-terminal domain. As reported previously, the positive control, native BSP5, bound to more than 95 % of the epididymal sperm cells [41]. Recombinant constructs AB and FL were found at the surface of ~50 % of the epididymal sperm. The other domains A, B and NA were also detected on sperm surface, but on fewer sperm (Not shown). The intensity of the signal for domains A and B was also weaker.

**Fig. 5:**
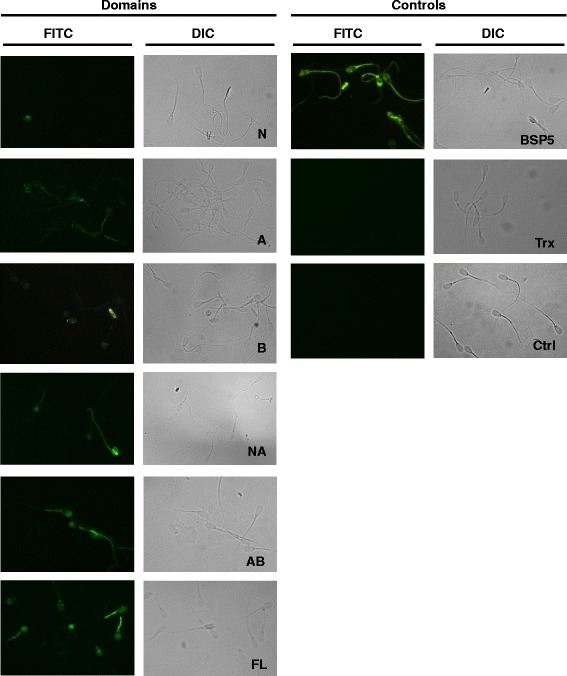
Immunostaining of recombinant BSP5 constructs on heparin-capacitated sperm. Sperm from cauda epididymis were incubated 20 min alone (Ctrl) or in media containing 60 μg/ml of each construct, 60 μg/ml Trx-His-S or 30 μg/ml native BSP5. Sperm were then washed and incubated for 5 h with 12 μg/ml of heparin. Aliquots of the samples were dried on poly-L-lysine slides. Slides were then incubated with anti-BSP5 or His-probe (Trx-His-S slide) antibodies at dilution of 1:100 and treated with FITC-conjugated IgG. (Original magnification × 630; DIC, Differential Interference Contrast)

To determine if the recombinant constructs and native BSP5 protein remain bound to sperm after capacitation processes, aliquots of sperm were taken at the end of the capacitation prior to the incubation with lyso-PC and evaluated by immunofluorescence (Fig. 5). Patterns observed were similar to those observed in non-capacitated conditions indicating that proteins were still present, and that localization of the domains on sperm surface did not change following capacitation. The binding of the recombinant constructs (A, B, NA, AB and FL), like the binding of native BSP5, was not restricted to head portion of the sperm as proteins were also localized to the midpiece of the sperm. No signal was obtained when sperm were incubated without protein (control) or with Trx-His-S using His-probe antibody as primary antibody.

## Discussion

Bovine BSP proteins are multifunctional proteins, via their interaction with cell membranes and with proteins such as GAGs. Heparan sulfate and other GAGs like heparin and chondroitin sulphate B are present in oviductal and follicular fluids of bovine species [28]. Since heparin is commonly used to induce the sperm capacitation in bovine species [26], it was of interest to check if the recombinant BSP5 protein constructs could bind to heparin and chondroitin sulphate B. It is not surprising that the N-terminal part of the protein did not show any interaction with the GAGs, as this domain is rich in negatively charged amino acids and does not contain the known heparin binding-consensus sequences B-B-X-B or B-X-X-B-X-B-B (where B represent basic residues and X represents any residues) [42, 27]. All the other recombinant constructs displayed expected binding characters with respect to heparin and chondroitin sulphate B. Results seem to suggest that glycosylation is not essential for the binding of bovine BSP proteins to heparin or chondroitin sulphate B. These results contradict the observation reported for porcine and equine BSP proteins. In these species, it seems that glycosylation can modulate the aggregation state of the BSP proteins and thus has an indirect effect on the ability of these proteins to bind to heparin [43].

Although gelatine binding is a characteristic feature of Fn2 domains, the biological relevance of this property in bovine BSP proteins is not known. Of all the domains tested in the current study, only the N-terminal domain failed to interact with gelatine. Results obtained are in accordance with previous experiments performed with BSP1 B domain isolated by tryptic digestion [44]. Interestingly, results seem to suggest that B domain has a greater affinity than domain A, even with the presence of the N terminal part in the NA construct. This slightly lower affinity of A and NA constructs could possibly be due to the presence of a phenylalanine residue in position 108 of the BSP5 protein sequence. Indeed some previous studies on the structure of Fn2 domains have shown that a tyrosine residue in this position is necessary for strong gelatine binding [45].

As expected, A, B, NA, AB and FL constructs, which contain one or two Fn2 domains, interacted with PC liposomes. This is in agreement with the established fact that the Fn2 domains are responsible for the binding to PC groups [46, 20]. The PC ligand binding is mediated by a cation- π interaction between the quaternary ammonium group of the choline moiety and the indole ring of a core tryptophan residue, and hydrogen bonding between the phosphate group and exposed tyrosine residues of the BSP proteins [20]. In contrast, the negatively charged N-terminal part did not interact with neutral lipid PC. As BSP proteins bind to sperm via an interaction with choline phospholipids, all recombinant domains interacting with PC liposomes also bound to bovine epididymal sperm.

In this study, we also demonstrated for the first time that the native BSP5 protein and all recombinant constructs, except the N domain, remained associated with sperm even after heparin-mediated capacitation. These results are in contradiction with the work previously published by Hung *et al.,* who observed a complete loss of BSP5 on sperm surface following 5 h-incubations in the presence and absence of heparin [47]. This discrepancy could be due to the different antibody used in each study. Hung *et al.,* used an antibody against an N-terminal peptide of the BSP5 protein, whereas the antibody used in the current study was prepared against the full-length native BSP5 protein. This difference between studies could suggest, that during sperm capacitation or during prolonged incubation, BSP5 is not lost, but is partly cleaved or modified at its N-terminal part.

After binding studies, the recombinant domains were tested for their ability to induce heparin-mediated sperm capacitation. As mentioned previously, the acrosome reaction induced by lyso-PC in the present context is meant to reflect capacitation. Recombinant AB domain and full-length BSP5 protein stimulate the capacitation to the level comparable to native BSP5 protein. The recombinant A, B, NA and N domains and the control Trx-His-S construct did not have any effect on the sperm capacitation mediated by heparin. These results are in agreement with results obtained by Moreau *et al.,* showing that the B domain of BSP1 is not sufficient to stimulate sperm cholesterol efflux associated with capacitation [44]. Since there is no difference in stimulation in the level of sperm capacitation observed in presence of the AB construct versus the full-length construct, it seems that the N-terminal part does not appear to play role in sperm capacitation. Similar functional mapping studies on the human ZP3, which acts as a primary sperm receptor, have concluded that the functional activity of the protein resides in its C-terminal domain [48]. Similar functional mapping studies of sperm proteins have also been used to define parts of proteins, which could be used as contraceptive antigen [49].

As reported previously for experiments with the native BSP5 protein [26], the spontaneous acrosome reaction remained low in the presence of all recombinant constructs. This suggests that similar to the native BSP5 protein, the recombinant constructs cannot stimulate the acrosome reaction by themselves, and induction by lyso-PC is essential for stimulation of the acrosome reaction of capacitated sperm. All these results tend to demonstrate that the domains A and B together constitute the minimal functional unit for sperm capacitation induced by heparin. Since recombinant protein constructs were expressed in *E. coli*, it became evident that glycosylation is not required for BSP binding to sperm or for its function in heparin-induced capacitation. It is therefore possible that the glycosylation of BSP proteins is not required for them to perform their function, but could play other roles. For example, mouse sperm protein IZUMO, a protein essential for sperm fusion with eggs, glycosylation is not required for the function but has a protective role [50].

## Conclusions

In conclusion, this study demonstrates that two tandem fibronectin domains at the C-terminus part of the protein are sufficient to mediate the sperm capacitation in bovine BSP5. The N-terminal part and glycosylation have no direct role in mediating sperm binding or capacitation or acrosome reaction. As the modular structure of BSP proteins is highly conserved among mammals, these results could be extended to other members of BSP superfamily. The function of N-terminal part still remains elusive. BSP proteins were discovered three decades ago, some new functions have been proposed in the recent years for these proteins. It will be interesting, to know, if the N-terminal part of the BSP proteins and/or its glycosylation is important for these new functions.
